# Right brain-to-right brain psychotherapy: recent scientific and clinical advances

**DOI:** 10.1186/s12991-022-00420-3

**Published:** 2022-11-19

**Authors:** Allan Schore

**Affiliations:** Department of Psychiatry and Biobehavioral Sciences, UCLA David Geffen School of Medicine, Los Angeles, CA USA

**Keywords:** Right brain, Affect regulation, Attachment, Intersubjectivity, Synchrony, Unconscious, Psychotherapy, Hyperscanning neuroimaging, Right temporoparietal cortex

## Abstract

This article overviews my recent acceptance of a Lifetime Achievement Award from Sapienza University of Rome, in which I discussed three decades of my work on the right brain in development, psychopathogenesis, and psychotherapy. In the following, I offer current brain laterality and hemispheric asymmetry research indicating that right brain emotional and relational processes operate beneath conscious awareness not only in early human development, but over the lifespan. I discuss recent interdisciplinary studies on the central role of ultrarapid right brain-to-right brain intersubjective communications of face, voice, and gesture and the implicit regulation of emotion in nonverbal attachment dynamics. Special emphasis is on the fundamental psychobiological process of interpersonal synchrony, and on the evolutionary mechanism of attachment, the interactive regulation of biological synchrony within and between organisms. I then present some clinical applications, suggesting that effective therapeutic work with “primitive” nonverbal emotional attachment dynamics focuses not on conscious verbal insight but on the formation of an unconscious emotion-communicating and regulating bond within the therapeutic relationship. Lastly, I review recent hyperscanning research of the patient’s and therapist’s brains during a face-to-face, emotionally focused psychotherapy session that supports the right brain-to-right brain communication model. I end suggesting that the right brain is dominant in both short-term symptom-reducing and long-term growth-promoting deep psychotherapy.

## Background

The background of this article is my recent acceptance of a Lifetime Achievement Award from Sapienza University of Rome, and my response to this honor, a presentation on the right brain and its central role in development, psychopathogenesis, and psychotherapy to a large psychiatry audience. In the following I’d like to briefly overview three decades of this work, offer some comments on what I view as the major current trends in psychotherapy practice and research, as well as what I see as changes and future directions of the mental health field. You’ll note that I frequently use the literal voices of scientists and clinicians to show convergence and common language now being used to describe the underlying psychobiological mechanisms central to the change processes in the mother–infant attachment and therapist–patient psychotherapy relationships.

In my first book *Affect Regulation and the Origin of the Self* published in 1994 (and re-released as a Classic Edition in 2016), I explored the neurobiology of human emotional development, concluding that affective processes, acting beneath levels of awareness, lie at the affective core of the subjective self [1]. The focus was on the early developing right brain in bodily-based attachment dynamics, in both early development and in psychotherapy. In a subsequent volume at the beginning of this century on affect dysregulation I discussed attachment trauma and the etiology of psychiatric and personality disorders [2], and in another on the treatment of affect dysregulation of the early developing emotional right brain [3]. By this time in “the decade of the brain” an “emotional revolution” was occurring in psychotherapy, and clinical models were moving towards brain–mind–body conceptualizations. In the books, articles, and chapters that followed I continue to offer new interdisciplinary evidence that right brain emotional processes beneath conscious awareness are operative not only in early human development, but over the lifespan. Indeed, over three decades I am suggesting that we are experiencing a paradigm shift from left brain conscious cognition to right brain unconscious emotional and relational functions.

Towards that end I continue to cite an expanding body of neurobiological and clinical studies indicating that the functional and structural differences between the two brain hemispheres is profound [4, 5]. A massive body of brain laterality studies describe in some detail how each cerebral hemisphere has a distinct mode of attending to the world, and creates coherent, utterly different and often incompatible versions of the world, with competing priorities and values. Due to current rapid advances in neuroscience, brain asymmetry, although once controversial, is now in agreement that different dual lateralized cortical–subcortical systems exist with unique structure–functions relationships (e.g., rational brain vs. emotional brain; linguistic brain vs. social brain; analytical vs. intuitive brain; explicit vs. implicit self systems; conscious vs. unconscious minds). In his classic volume *The Right Brain and the Unconscious: Discovering the Stranger Within*, the clinical neuropsychologist Rhawn Joseph [6] observed,

Just as we have a conscious and an unconscious mind, as well as a right and left brain, we also have two self-images. One is consciously maintained and the other is almost wholly unconscious. The *conscious self-image* is associated with the *left half of the brain* in most people. However, this self-image is also subject to unconscious influences. By contrast, the *unconscious self-image* is maintained within the *right brain* mental system and is tremendously influenced by current and past experiences… the two self-images… interact. Indeed, sometimes the conscious self-image is fashioned in reaction to unconscious feelings, traumas, and feared inadequacies that the person does not want to possess, but that nevertheless, are unconsciously maintained.

**Fig. 1:**
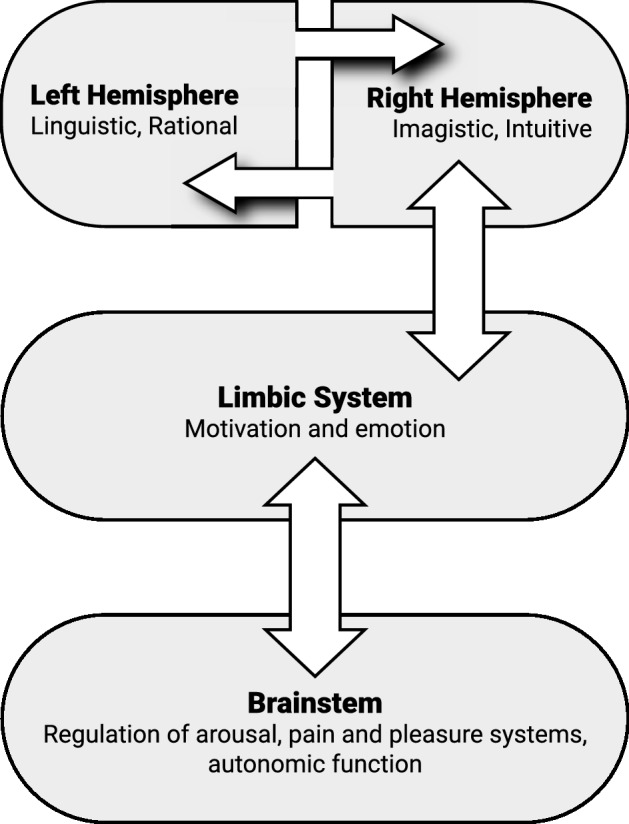
Unconscious processing of the “lower”, “bottom up” early developing implicit right brain and subsequent connections into the “higher”, “top down” later developing conscious explicit left brain. Note the vertical axis of the emotional right brain on the right side of the figure

Continuing this theme, the neurologist Guido Gainotti [7] offered an article on “Emotions, Unconscious Processing and the Right Hemisphere”, where he concluded, “The right hemisphere may subserve the lower schematic’ level (where emotions are automatically generated and experienced as ‘true emotions’) and the left hemisphere the higher ‘conceptual’ level (where emotions are consciously analyzed and submitted to intentional control.” More recently, the neuropsychiatrist Iain McGilchrist [8] asserts,

If what one means by consciousness is the part of the mind that brings the world into focus, makes it explicit, allows it to be formulated in language, and is aware of its own awareness, it is reasonable to link the conscious mind to activity almost all of which lies ultimately in the left hemisphere. The right hemisphere both grounds our experience of the world at the bottom end, so to speak, and makes sense of it, at the top end…this hemisphere is more in touch with both affect and the body…neurological evidence supports what is called the primacy of affect and the primacy of unconscious over conscious will (see Fig. 1).

Another central area of my work is in neuropsychoanalysis, the science of unconscious processes, where I contend that the right brain is the psychobiological substrate of human unconscious mind first described by Sigmund Freud. Recent authors are now describing a right hemispheric dominance in nonconscious processing, concluding “The right hemisphere has an advantage in shaping behavior with implicit attention whereas the left hemisphere plays a greater role in expressing explicit knowledge” [9]. Indeed there is now agreement that implicit processing is equated with unconscious processing (see [4, 5, 10]. Recently Ladavas and Bertini offer an article on “Right hemisphere dominance for unconscious emotionally salient stimuli”, where they describe the “specialization of the right hemisphere in the processing of emotional stimuli occurring outside the focus of awareness” [11].

In 1994 I proposed that the right brain is dominant in maintaining an unconscious cohesive, continuous, and unified sense of self [1]. I further suggested that in two-person attachment dynamics the bodily-based subjective self intersubjectively communicates its emotional states nonverbally, right brain-to-right brain, with another subjective self. Just as the left brain communicates its states to other left brains via conscious linguistic behaviors, so the right nonverbally communicates its unconscious self states to other right brains that are *sensitively tuned to receive these intersubjective salient emotional communications*. Following this up in my 2003 volumes I stated in contrast to a static deeply buried storehouse of ancient memories buried and silenced in “infantile amnesia”, contemporary psychoanalysis now refers to a *“relational unconscious*”, whereby one unconscious mind intersubjectively communicates with another unconscious mind [2, 3]. This model harkened back to Freud’s assertion at the beginning of the last century, “It is a very remarkable thing that the *Ucs* of one human being can react upon that of another, without passing through the *Cs*”, [12] and the pioneering work of the Hungarian psychoanalyst Sandor Ferenczi, who first described his concept of an intersubjective dialogue between one unconscious and another unconscious [13].

The interpersonal neurobiological construct of a relational unconscious is the most radical transformation of Freud’s psychoanalytic theory. Updated reformulations of the unconscious have shifted from an *intrapsychic* unconscious that expresses itself in dreams at night to an *interpersonal* relational unconscious, in which the unconscious mind of one communicates with the unconscious mind of another, and is omnipresent in everyday life. In parallel writings to my own, Karlen Lyons-Ruth offered a “two-person unconscious” asserting, “Most relational transactions rely heavily on a substrate of affective cues that give an evaluative valence or direction to each relational communication. These occur at an implicit level of rapid cueing and response…too rapidly for simultaneous verbal transaction and conscious reflection” [14]. I would add that these communications emerge in early infancy, shaping the structural and functional development of the unconscious mind’s right brain survival functions. Indeed, implicit right brain-to-right brain intersubjective nonverbal communications are expressed in attachment dynamics at unconscious levels for the rest of lifespan.

### Right brain-to-right brain communications in early human development

At the core of my developmental work on intersubjectivity and attachment is the central principle of interpersonal neurobiology: the self organization of the developing brain occurs in the context of a relationship with another brain, another self [2]. Utilizing an interdisciplinary perspective, regulation theory models the underlying mechanisms by which the structure and function of the mind and brain are shaped by early experiences, especially emotional experiences, as well the relational mechanisms by which communicating brains intersubjectively *synchronize*, align, and *couple* their neural activities with other brains. The term synchrony derives from Greek syn, same or common, and chronos, time, and thereby means occurring at the same time, in the same moment, and thus simultaneous. There is now agreement that the process of interpersonal synchrony acts as a primal social bonding mechanism, and that early synchronous shared social interactions are the foundation of the human experience [1, 4, 15].

**Fig. 2:**
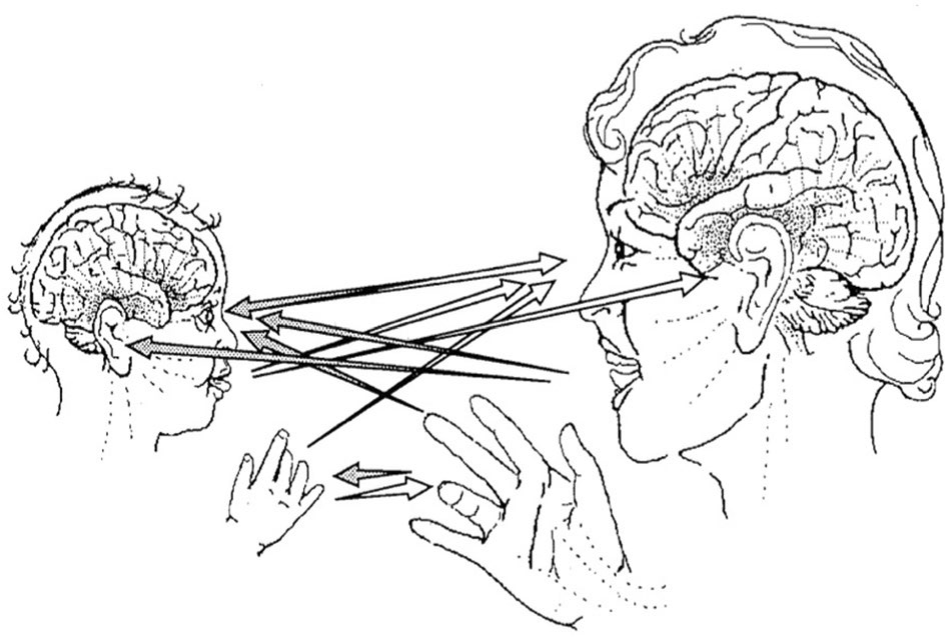
Channels of face-to-face communication in primary intersubjectivity. Protoconversation is mediated by synchronized eye-to-eye orientations, vocalization, and hand gestures all acting in coordination to express interpersonal awareness and emotions. From Trevarthen [16]

In classic research Colwyn Trevarthen documented the early origins of human intersubjectivity at 2–3 months, when infants are ready to engage in behavioral turn-taking and expect social contingency and predictable back-and-forth interactivity [16]. He observed visual (mutual gaze), auditory, and tactile playful, affectionate emotional communications in which the intuitive mother and her infant, intently looking and listening to each other, bidirectionally synchronize and mutually regulate their emotional states. In such “protoconversations” positive emotions of both members of a dyad are expressed and actively perceived in spontaneous, reciprocal, rhythmic turn-taking interactions (see Fig. 2).

According to Trevarthen, this two-way traffic of intersubjective visual, auditory, and tactile signals induces instant positive emotional effects, namely excitement and pleasure build within the dyad. These collaborative emotional transactions trigger interpersonal resonance within the emotionally communicating dyad, thus generating the intercoordination of *synchronized* and thereby *shared positive affective brain states*. But his model also focuses on internal structure–function events, where he states that the intrinsic regulators of human brain growth in a child are specifically adapted to be *coupled*, by emotional communication, to the regulators of adult brains.

In a recent article on the interpersonal neurobiology of intersubjectivity I have cited a recent body of brain asymmetry studies to argue that Trevarthen’s synchronized intersubjective protoconversations represent rapid, reciprocal, bidirectional visual–facial, auditory–prosodic, and tactile–gestural right brain-to-right brain implicit nonverbal communications between the mother and her developing infant [17]. In such I emphasize the essential functions of right temporoparietal junction (right TPJ) in the posterior sensory areas of the developing right hemisphere in sending and receiving these emotionally charged imagistic nonverbal communications. The right TPJ, a central hub of the right-lateralized social brain, integrates input from visual, auditory, somesthetic, and emotional limbic areas. For the rest of the lifespan this system plays a pivotal locus in self functions: face and voice processing, perceptual awareness, collaborative social interactions, and the representation of subjective emotional experience.

Soon after, the neurologist Kaisa Hartikainen [18] writing on “emotion–attention interaction in the right hemisphere” stated,

The right TPJ has been suggested to be a central hub for…non-verbal emotional communication and interaction between a caregiver and an infant (Schore, 2021). This caregiver-infant pre-verbal prosodic, gestural, and facial emotional expressions, provides a basis for the development of attachment…Successful emotional communication and downregulation of infant’s negative emotions relies on right hemispheric functions of both the caregiver and infant.

Furthermore, the right TPJ is also involved in “emotional arousal linked with positive emotion (Schore)”, and in “the synchronization between the brains of two people.”

In classic writings, John Bowlby proposed attachment communications are accompanied by the strongest of feelings and emotions, and, like Trevarthen suggested they occur within a context of facial expression, tone of voice, and posture [19]. A large body of research supports what De Heering and Rosson call “rapid categorization of natural face images in the infant right hemisphere” [20]. In an overview of research on the laterality of the “human social brain” Brancucci and colleagues conclude, “The neural substrates of the perception of voices, faces, gestures, smells, and pheromones, as evidenced by modern neuroimaging techniques, are characterized by a general right-hemispheric functional asymmetry” [21]. At levels beneath awareness, humans tend to display right hemispheric “left gaze bias”, whereby they direct their initial gaze to the left side of the other’s face, and look longer exploring the left side, which is more expressive [22].

Indeed, the essential task of first two years of life is the cocreation of an intersubjective right brain-to-right brain attachment bond of emotional communication and interactive regulation between the infant and primary caregiver. Secure attachment occurs via the mother’s attention and implicit “background presence” of synchronized attunement, recognition, and regulation not of the infant’s voluntary behavior, but with moment-to-moment alterations of right brain autonomic involuntary emotional arousal, the physiologic dimension of child’s affective state. Hartikainen observes a central role of the right hemisphere in attention, emotion, and arousal [18].

The research of Manini’s laboratory reports that synchronization of the mother’s responses to infant signals in their dyadic interaction is a central aspect of sensitive parenting, because it directly relates to the promptness of the mother’s response and her adaptation moment by moment to the child’s emotional state [23]. This embodied and pre-reflective sensitivity allows the mother to immediately recognize any shift in the child’s emotional needs, as well as promptly soothe the child when distressed. These synchronized interactions enable the psychobiologically attuned mother and child to become sensitive to each other’s physiology and behavior, and thereby the formation of a unique bond between them. They conclude, the autonomic nervous system represents an elementary mechanism supporting emotional synchrony between mother and infant. More recently Wass et el. observe that parents mimic and influence their infant’s autonomic activity through dynamic affective state matching [24]. They document changes in infant arousal lead to autonomic changes in the parent, and that moments when the adult showed greater autonomic reactivity were associated with faster infant quieting.

That said both research and clinical evidence indicate that the primary caregiver is not always attuned, that there are frequent moments of stressful misattunement in the dyad, ruptures of the attachment bond. A major attachment process is expressed in “interactive repair” following misattunement, in which the caregiver who induces a stress response, in a timely fashion spontaneously reinvokes a re-attunement and regulates the infant’s negatively charged emotional arousal [1, 15]. This dyadic mechanism allows for the reestablishment of interpersonal synchrony after an asynchrony between them. It also generates trust in the infant that the caregiver will be emotionally available at times of stress. Rupture–repair is common in secure but not insecure–avoidant, insecure–resistant, or especially disorganized attachment mother–infant dyads.

In 2008 my wife Judith and I published an article “Modern Attachment Theory”, where we suggested a body of experimental and clinical data on how affective bodily-based processes are nonconsciously interactively regulated had shifted attachment theory to a regulation theory [25]. I use the term regulation theory in order to explicitly denote that I am offering a theory, a systematic exposition of the general principles of a science. Specifically, it is an interdisciplinary formulation of the central psychobiological processes that underlie early human emotional and social development, one that formulates research hypotheses that are experimentally testable and clinically applicable. At the core of the theory the developmental processes of intersubjectivity represents the *communication* of emotion, while attachment represents the *regulation* of states of affective arousal. Thus, the evolutionary mechanism of attachment represents the regulation of biological synchrony *within and between* organisms. It is accessed by the secure mother to implicitly track and regulate the infant’s emotional arousal. A central tenet of the theory dictates that the structural organization of attachment circuits self-organize in an early critical period of growth of the emotional right brain from the last trimester of pregnancy until the third year when the left begins one, and that the infant’s anterior right orbitofrontal (ventromedial) cortex, the apex of limbic system, matures over this period. This right-lateralized system acts as attachment control system of effortless, subliminal, implicit affect regulation.

In the most comprehensive study to date on early right brain development, Bosch-Bayard and colleagues offer a study in *NeuroImage*, “EEG effective connectivity during the first year of life mirrors brain synaptogenesis, myelination, and early right hemisphere predominance” [26]. These authors measured the connectivity of different areas of the infant brain at 2–3, 5–8, 8–12 months, and show an asymmetric lateralized increase in specifically the right and not left hemisphere. They conclude,

The right hemisphere is predominant during the preverbal epoch in infants…and lasts during the first three years of life (Schore, 2000). The right hemisphere is understood as an executive regulatory system of the emotional brain involved in inhibitory control. In particular the right orbital prefrontal region acts as an executive control for the entire right brain (Schore, 2000). The right predominance starts shifting to the left hemisphere by the age of 3 years.

Over the first two years of human infancy the growth and lateralization of infant’s early developing right hemisphere is thus dependent upon the implicit safe and trusting emotional interactions generated in the mother–infant attachment relationship [27].

Studying brain development at the beginning of the first year, developmental neuroscientists conclude, “In early life the right cerebral hemisphere could be better able to process... emotion (Schore). This idea appears consistent with our findings of rightward asymmetry in limbic structures. These neural substrates function as hubs in the right hemisphere for emotion processes and mother and child interaction” [28]. Tronick’s group reported that in the middle of the first year 6-month-old infants use left-sided gestures generated by the right hemisphere in order to cope with the stressful still-face paradigm. These data are “consistent with Schore’s hypotheses of hemispheric right-sided activation of emotions and their regulation during infant–mother interactions and his argument that the left side of the brain is less developed than the right side” [29]. Minagawa-Kawai and colleagues studying infant–mother attachment at end of first year conclude “Our results are in agreement with that of Schore who addressed the importance of the right hemisphere in the attachment system” [30].

Furthermore, the mother–infant attachment relationship impacts the developing right brain for better or worse. It can either facilitate a healthy resilience to stress or create a vulnerability to characterological affect dysregulation and deficits in social relationships, and thereby later psychopathology. In the right brain critical period what I have termed “relational trauma”, chronic attachment trauma that results from repeated and prolonged exposure to highly dysregulated early relational and emotional experiences without repair induces disorganized insecure attachments that imprint a physiological reactivity and susceptibility to later disorders of affect regulation [2]. These misattuned mothers frequently fail to coordinate and synchronize with their infant’s emotional states. Such implicit preverbal attachment dynamics are represented in the early developing emotional right brain as an imprinted unconscious insecure internal working model of attachment, before left hemispheric maturation.

Fifty years ago Bowlby [31] suggested,

When multiple models of a single figure are operative they are likely to differ in regard to their origin, their dominance and the extent to which the subject is aware of them. In a person suffering from an emotional disturbance, it is common to find that the model that has had greatest influence on his feelings and behavior, is one developed during his early years and is constructed along fairly primitive lines, but that the person may be relatively unaware of while simultaneously there is operating within him a second, and perhaps radically incompatible model that developed later, that is much more sophisticated, and that the person is more clearly aware of and he may mistakenly assume to be dominant.

Modern attachment theory offers an early unconscious right brain preverbal model of implicit “primitive” emotional attachment dynamics, while “classical”, “academic” attachment theory describes the explicit conscious behavioral/cognitive functions of the later-forming verbal left brain. The Oxford English dictionary defines primitive as “of or pertaining to the first age, period, or stage; early, ancient.”

### Clinical applications of regulation theory

The right brain attachment dynamic is a central focus of regulation theory, and affect dysregulation plays a critical role in the both the symptomatology and treatment of all psychiatric and personality disorders. In my studies I offer interdisciplinary and clinical evidence indicating that the co-constructed psychotherapy relationship itself plays a major role in symptom-reducing and growth-promoting treatment, and that the right hemisphere is dominant in psychotherapy [5, 15]. Effective clinical work with “primitive” nonverbal emotional attachment dynamics of the first foundational years of life focuses not on verbal cognitive insight but on the formation of an emotion-communicating and regulating bond between the patient and the empathic clinician.

This conceptualization attends to two fundamental questions—how do we work directly with the patient’s and our own emotions, and how do we access “primitive” nonverbal intersubjective emotional communications within the psychotherapy session? In any session the empathic therapist is consciously, explicitly attending to the patient’s verbalizations in order to objectively diagnose and rationalize his or her dysregulating symptomatology. However, the therapist is also intersubjectively listening and interacting at another level, an experience-near subjective level, one that implicitly processes the patient’s implicit moment-to-moment nonverbal bodily-based emotional communications at levels below awareness, beneath the words.

In “heightened affective moments” of a session the empathic therapist “follows the patient’s affect”, and transiently callosally shifts out of the left brain into a right brain state of wide-ranging evenly suspended attention [1, 5, 15]. In this bodily-based therapeutic interaction the “sensitive” clinician intuitively and fluidly tracks and matches the patient’s rhythmic moment-to-moment crescendos and decrescendos of emotional arousal and changes in affective states. Recall, the right brain is dominant for arousal [18]. In 1994 I suggested that as in the secure mother’s attachment relationship, in therapy the clinician’s right orbitofrontal cortex implicitly tracks the patient’s dynamically changing emotions [1].

In 2012 Goodkind and colleagues published “Tracking emotional valence: the role of the orbitofrontal cortex” in *Human Brain Mapping* [32]. These researchers demonstrate that the right orbitofrontal cortex is involved in continuously tracking dynamically changing emotions, “enabling us to understand the emotions expressed by others in real time, follow them as they unfold and change, and adjust our behavior in ways that are appropriate.” These data support my assertion that the psychobiologically attuned therapist decodes the bodily-based nonverbal communications of the patient’s right brain by interoceptive actual felt emotional reactions, and thereby a form of empathic responding. The intuitive clinician is implicitly learning the rhythmic structures of patient’s internal states, and modifying her behavior to synchronize and couple with that structure, right brain-to-right brain. This interpersonal synchrony, expressed in a *coupling of the therapist’s and patient’s right brain*s, also enables the patient’s embodied subjective self to implicitly experience “feeling felt” by the empathic therapist.

Writing on “Physiological synchrony in psychotherapy sessions” Tschacher and Meier [33] state that synchrony between therapist and patient is expressed in their central and autonomic nervous systems moving in a synchronized way over time. They observe, “Synchrony is generally defined as the social coupling of two (or more) individuals in the here-and-now of a communication context that emerges alongside, and in addition to, their verbal exchanges*.*” Note this this positional reference describes the *nonverbal right hemisphere and coupled right brains*. This right-lateralized psychobiological system intersubjectively synchronizes and couples with another “emotional” right brain that is “attuned” and “on the same wavelength.” This right brain-to-right brain coupling allows the empathic therapist to share and regulate the patient’s subjective affective states, especially during stressful transferential–countertransferential clinical reenactments of early attachment trauma within the therapeutic relationship, common in the treatment of early forming personality disorders [3, 5, 15].

With direct implications for the therapeutic relationship Decety and Chaminade observe, “Mental states that are in essence private to the self may be shared between individuals…self-awareness, empathy, identification with others, and more generally intersubjective processes, are largely dependent upon…right hemisphere resources, which are the first to develop” [34]. More recently McGilchrist writes, “the social and empathic self, and the continuous sense of self, with ‘depth’ of existence over time, is more dependent on the right hemisphere”, concluding, “Without a self, there is no capacity for intersubjectivity, for the experience of shared time and shared space” [10]. In parallel classical writings in the psychodynamic psychiatry literature Whitehead [35] asserts,

Every time we make therapeutic contact with our patients we are engaging profound processes that tap into essential life forces in our selves and in those we work with. *Emotions are deepened in intensity and sustained in time when they are intersubjectively shared*. This occurs at moments of ‘deep contact’ (italics added).

Similarly, the neuropsychologist Julian Keenan and colleagues state, “The right hemisphere, in fact, truly interprets the mental state not only of its own brain, but the brains (and minds) of others” [36]. In my 2012 volume *The Science of the Art of Psychotherapy* I suggested that across disciplines we were witnessing a paradigm shift from a one-person intrapsychic to a two-person relational psychology, a shift in perspective from *within* a brain to an intersubjective relationship *between* brains, such as the right brain-to-right brain mother–infant attachment and therapist–patient psychotherapy relationships [15].

In a recent comprehensive overview of studies of the psychotherapy relationship Norcross and Lambert [37] conclude,

Decades of research evidence and clinical experience converge: the psychotherapy relationship makes substantial and consistent contributions to outcome *independent of the treatment*…We need to proclaim publicly what decades of research have discovered and what hundreds of thousands of practitioners have witnessed: The relationship can heal…What does not work are poor alliances in adult, adolescent, child, couple, and family psychotherapy as well as low levels of cohesion in group psychotherapy. Paucity of collaboration, consensus, empathy, and positive regard predict treatment dropout and failure.

Another large body of psychotherapy research focuses on the critical role of “common factors”, qualities of “effective therapists” associated with successful treatment outcomes, such as collaboration, empathy, conveyed respect, acceptance, genuineness, maintaining a warm emotional bond with the patient, and capacities for alliance building [38, 39]. Moreso than left brain semantics, each of these essential functions are expressed in the clinician’s nonverbal right brain as it is co-creating the therapeutic working alliance with the patient (see 3, 5, 15, 40). “Common factors” thus represent right brain reparative mechanisms that can be accessed in all forms of psychotherapeutic treatment.

Over the last three decades I continue to offer brain laterality research and clinical data on working right brain-to-right brain in the therapeutic alliance. These spontaneous nonverbal communications of self states take place in the present moment, a time frame of thousandths of a second to 2 to 3 s. My colleague Russel Meares referred to a form of therapeutic conversation that can be conceived as a dynamic interplay between two right hemispheres [41]. The relational psychoanalyst Philip Bromberg observed, *“*Allan Schore writes about a right brain-to-right brain channel of affective communication—a channel that he sees as ‘an organized dialogue’ comprising ‘dynamically fluctuating moment-to-moment state sharing. I believe it to be this process of state sharing that allows ‘a good psychoanalytic match” [42]. Note the direct allusion to the therapist and patient being “in sync”.

According to Koole and Tschacher interpersonal synchrony establishes interbrain coupling that provides “patient and therapist with access to another’s internal states, which facilitates common understanding and emotional sharing” [43]. Writing on brain-to-brain coupling as a mechanism for creating and sharing a social world, Hasson suggests, “Brain-to-brain coupling is analogous to a wireless communication system in which two brains are coupled via the transmission of a physical signal (light, sound, pressure or chemical compound) through the shared physical environment” [44]. Note this describes the implicit visual, auditory, and tactile nonverbal emotional transmissions shared face-to-face between two right brains communicating at ultrarapid rates, and thereby “hidden in plain sight” and invisible to the left hemisphere. A body of research indicates that the right occipital-temporal cortex generates a holistic face representation in only 170 ms, beneath conscious awareness [45]. Both research and clinical studies document that states shared between two individuals in both development and psychotherapy occur via synchrony, and that this fundamental developmental mechanism underlies emotion transmission, affective reciprocal exchange, physiological linkage, and empathy, all right brain relational functions.

Kaiser and Butler [46] now assert that in relational systems successful engagement is expressed in automatic and implicit sharing of social content, including emotions, where two or more persons understand the world “more or less as one”:

The implicit sharing process is temporal and bidirectional between…people…a mutual dynamic process is occurring, whereby partners make micro-adjustments over time driven by implicit information from high-resolution perceptions of the others’ states and intentions (Schore, 1994/2016). Mutual interaction…involves a complex fitting-together of the individuals involved, producing a resonance between two attuned systems and feelings of psychological closeness (Schore, 1994/2016).

This fitting together of an emotional engagement is the outcome of a synchronized, intersubjective, nonverbal dialogue embedded in a collaborative right brain-to-right brain communication system at the deep psychobiological core of the therapeutic alliance.

### Right brain-to-right brain communication—update

In 1994 my right brain-to-right brain communication model was based on theoretical principles, clinical explorations, and extant neuroscience research. Even more direct evidence of the “two-person”, “two-brain” model awaited an emergent technology that could simultaneously measure two brains interacting face-to-face in real time. Over the last decade “hyperscanning” methodologies utilizing electroencephalography, near-infrared spectroscopy, functional magnetic resonance, and magneto-encephalography providing simultaneous measurements of two individuals are now available. This technological advance allows for the study of two brains in real time social interactions with each other, during rapid bidirectional nonverbal communications.

Inspired by the developmental research of Beebe, Tronick, and especially Trevarthen on the intersubjective nonverbal communication and coordination between a mother and her infant (see Fig. 2), Dumas and his colleagues offered a now classic dual EEG hyperscanning study of a spontaneous nonverbal social interaction between two adults, characterized by reciprocal communication and turn-taking [47]. This *“two-body”* methodology allows for a simultaneous measurement of brain activities of each member of a face-to-face dyad during a two-way intersubjective nonverbal communication, where “both participants are continuously active, each modifying their own actions in response to the continuously changing actions of their partner.”

**Fig. 3:**
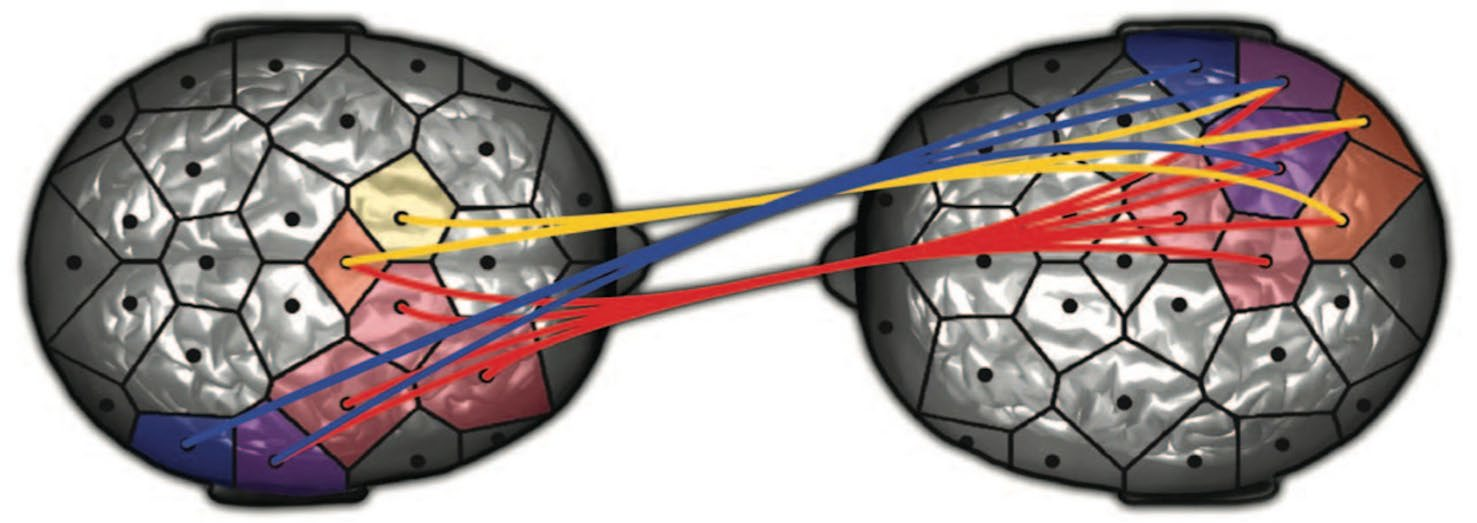
Top-down view of right-lateralized interbrain synchronization of a spontaneous bidirectional nonverbal communication. Adapted from “Toward a Two-Body Neuroscience.” From Dumas [48]

These researchers report changes in both brains in this relational context where both share attention and compare cues of self and other. Furthermore they document an interbrain synchronization, on *time scale of milliseconds*, of right centroparietal regions, a neuromarker of social coordination in both interacting partners, as well as a synchronization between posterior right temporoparietal junction (right TPJ) of one partner and right TPJ of the other. They cite studies showing that the right cortical TPJ is activated in social interactions, empathic understanding, and making sense of another mind, all outside conscious awareness. The following top-down view shows this right-lateralized interbrain synchronization during a nonverbal communication. Note what I have termed a *right brain-to-right brain* interaction between one subjective self and another subjective self, whereby one unconscious mind intersubjectively communicates with another unconscious mind across an intersubjective field (see Fig. 3).

More recently, in a pioneering and groundbreaking work Zhang and her colleagues published in *Psychiatry Research Neuroimaging* the first hyperscanning study of simultaneous recordings of a patient’s brain and a therapist’s brain during a face-to-face psychotherapy session in the laboratory [49]. In a non-invasive functional near-infrared spectroscopy (NIRS) investigation entitled, “Interpersonal brain synchronization associated with working alliance during psychological counselling”, college students presented with both problems in emotions and interpersonal relationships. The researchers report that clinicians focused empathically on the patient’s “emotional states”, and in this “reciprocal communication both members observed each other’s nonverbal cues, facial expressions and gestures.” Therapists reported being attentive to “moment to moment cues” of “emotional expression and body posture”, and offered emotional feedback.

During the 40-min first counseling session the researchers observed increases in cortical blood flow (hemoglobin oxygenation) and interpersonal brain synchronization of the right temporoparietal junction (right TPJ) in both the clinician’s and patient’s brains. This increased right-lateralized interbrain synchronization was specifically related to subjective ratings of the affective “bond or positive personal attachments between dyads.” The authors interpreted these findings as showing that the clinician and client form an interbrain synchronization that plays an essential role in building a working alliance and a positive therapeutic relationship, and that this brain-to-brain coupling facilitates the development of an emotional bond in the therapeutic alliance in the first session. They conclude that that this important nonverbal skill improves the working alliance, and that training should now focus on how to effectively synchronize with clients.

In a more current publication this laboratory offered another functional near-infrared spectroscopy study “Experience-dependent counselor–client brain synchronization during psychological counseling” [50]. Here they use NIRS hyperscanning of the patient and therapist to study the role of the counselor’s experience in building an alliance with clients. Working with a similar clinical population as before, during the first counseling session the therapeutic dyad in a face-to-face context focused on nonverbal cues of each other and on bodily-based emotional states. They report that experienced therapists (psychologists) with 600–4000 h of clinical experience utilized an “integrative” clinical approach focusing on empathizing and offering emotional feedback to the client. These expert clinicians used “moment-to-moment cues (e.g., emotional expression, body posture) and tried to be attentive to their clients’ reactions.”

This laboratory again documented a counseling-induced right-lateralized interpersonal synchronization, a right TPJ-to–right TPJ alignment that was especially evident with clinicians who had more psychotherapy experience. The researchers show that in the first session the clinician’s ability to specifically focus on the client’s emotional states and to interpersonally synchronize with her or him is an expression of therapeutic expertise. They concluded that increased right-lateralized interbrain synchronization over the session is associated with tighter interpersonal closeness/connectedness and better alliance/emotional interaction, and that this study confirms an interpersonal synchrony model of psychotherapy which dictates that “the more tightly the client and counselor’s brains are coupled, the better the alliance” [43].

In both Zhang studies during the session synchronous brain activity is seen in an alignment of metabolic physiological activity of the right temporoparietal junction of both members of the therapeutic dyad. This patient–therapist right-lateralized brain synchronization is the same as the Dumas’ “two-body” nonverbal intersubjective communication (see Fig. 3). As in that nonverbal context, the right TPJ of the expert clinician is interpersonally synchronized with the right TPJ of client, thus forming a right brain-to-right brain emotion communication system that operates between the therapist and patient, beneath awareness of both [1, 3, 5]. Note these hyperscanning studies directly support the clinical principles that the establishment of an effective relationship is the most important criterion for measuring therapeutic expertise [51], and that expertise is expressed in the clinical ability to establish an effective relationship with various types of patients [52].

Overviewing current “multi-brain neuroimaging” and “hyperscanning” data, Ray and his colleagues [53] assert that among all forms of interbrain communications, the communication of emotion is the most important process for mental health. Note their emphasis on right brain emotion and not left brain cognition in treatment, and my earlier thoughts about a paradigm shift from conscious cognition to unconscious emotion as the major vector of therapeutic change. With respect to psychopathology, they argue that the interpersonal perspective of between-brain functional connectivity can allow for a deeper understanding of the relational deficits of depression, autism spectrum disorders, schizophrenia, personality disorders, social anxiety disorder, somatic symptom disorder, eating disorders, sexual dysfunctions, and suicide. Furthermore, they see the direct application of this paradigm shift in interbrain neuroimaging to the therapeutic alliance, defined as the collaborative bond between patient and therapist.

In offering the same Dumas hyperscanning image as above of coupled, synchronized right brains, Ray points out that all mental disorders unfold in an interpersonal context, and that in most social settings, the human brain works in interaction with other brains establishing a “coupling” between themselves. They conclude, “At present functional neuroimaging is on the brink of a paradigm shift, quantifying the *brain interactions between individuals transcending the boundary of the skull*.” Recall my earlier assertion that we are now experiencing a paradigm shift in psychotherapy from a one-person intrapsychic into a two-person interpersonal perspective (and thus ultimately an integrated model of both). In such synchronized interactions nonverbal relational and emotional therapeutic change mechanisms are activated in a face-to-face dialogue of two coupled right brains across an affectively energized *intersubjective field* (see Fig. 3).

Recent authors are now suggesting a “two-body approach” that captures interbrain synchronization, unconscious mutual attunement, and the dynamic exchange between individuals represents the *“dark matter”* of live social interaction [54]. Indeed, the right hemisphere has literally been described as “*the dark side of consciousness”* [36]. In total the brain laterality and hemispheric asymmetry research I’ve cited throughout this talk strongly support my work on a relational unconscious, an omnipresent ultrarapid and thereby invisible right brain-to-right brain communication system in the psychotherapeutic relationship, and on an affectively focused model of psychotherapy, including psychodynamic psychotherapy and psychodynamic psychiatry. I suggest that psychiatry should consider expanding its clinical models from exclusively focusing on conscious left brain verbal anxiety and depression symptomatology to also include unconscious emotional and relational deficits of the right brain. The ability to co-create a right brain-to-right brain relationship with different clinical populations directly impacts the psychiatrist’s skill in forming a bond of safety and trust with a variety of patients, in reducing treatment dropout, and in facilitating stronger drug compliance.

In 2014 I published an article in *Psychotherapy*, the flagship journal of the American Psychological Association Division of Psychotherapy, “The right brain is dominant in psychotherapy”, wherein I offered interdisciplinary evidence indicating that psychotherapy, “a relationship of care”, can alter more than the patient’s left-lateralized conscious mind. It also directly influences the growth and development of the unconscious “right mind” [55]. Note only a right and not left brain therapeutic approach can change the patient’s unconscious self image and unconscious internal working model of attachment. In recent clinical applications of regulation theory I have focused on autistic spectrum disorders [56], group psychotherapy [57], working with pathological dissociation [58], and therapeutic mutual regressions in reenactments of early attachment trauma [40]. And so I continue to explore the critical role of right brain emotional and relational processes in psychotherapy and psychoanalysis, asserting that the right brain is dominant in short-term symptom-reducing and long-term growth-promoting deep psychotherapy. In both, the psychotherapist’s relational and emotional expertise in working in psychotherapeutic relationships with a wide variety of patients, more than a mastery of techniques, lies at the core of the art of psychotherapy.

And I thank you for both your attention and for the great honor of this award.

## Data Availability

Availability of data and materials came from established journals in the field of neuroscience. Data sharing is not applicable as no data sets were generated or analyzed.

## Abbreviation

TPJ: Temporoparietal junction
